# A three-factor benefits framework for understanding consumer preference for scented household products: psychological interactions and implications for future development

**DOI:** 10.1186/s41235-022-00378-6

**Published:** 2022-04-01

**Authors:** Rachel S. Herz, Maria Larsson, Rafael Trujillo, Marisa C. Casola, Farah K. Ahmed, Stacy Lipe, Morgan E. Brashear

**Affiliations:** 1 Department of Psychiatry and Human Behavior, Alpert Medical School, Brown University Medical School, 146 Thayer St., Providence, RI 02912 USA; 2 Department of Psychology and Neuroscience, Boston College, Chestnut Hill, USA; 3 Department of Psychology, Stockholm University, Stockholm, Sweden; 4 The Procter & Gamble Company, Cincinnati, OH USA; 5 Fragrance Creators Association, Arlington, VA USA

**Keywords:** Scent, Olfaction, Emotion, Memory, Perception, Cognition, Motivated behaviour, Social behaviour, Confidence, Health, Quality of life, Scent marketing, Sensory marketing, Cross-modal interactions, Household consumer products, Consumer behaviour, Three-factor framework

## Abstract

Humans have deliberately scented their environment for purpose or pleasure for millennia. In the contemporary marketplace most consumers prefer and purchase scented versions of common household products. However, the drivers of this consumer preference have not been elucidated. To explain the attraction to scent in household products we propose a novel three-factor framework, comprising *functional benefits* (malodor mitigation, base odor coverage, freshening), *in-use experience benefits* (cleanliness, efficacy, pleasure), and *emotional benefits* (increasing in confidence, mood and nostalgia). To support this framework, we present new data from a market research survey on US consumer purchasing habits and attitudes towards home cleaning, laundry, and air freshening products. Further substantiating our framework, a focused review of olfactory psychological science illustrating the central role of scent in cognition, wellbeing, motivated behavior, and social behavior, as well as sensory marketing research highlights the benefits and implications of scent in consumer household products. Based on our three-factor framework we go on to discuss the potential for scent to influence health and raise issues to consider (such as potential negative responding to fragranced products). We conclude by showcasing new opportunities for future research in olfactory science and on scented household products that can advance the positive impacts of scent.

## **Significance statement**

This paper provides a framework for understanding the importance of scent in everyday consumer products, daily activities and quality of life. While scent use in the household has a long cultural history, it is occasionally criticized as an unnecessary luxury. Our proposed three-factor benefits framework (*Functional, In-Use Experience, Emotional*) explains consumer preferences for scented products such as laundry treatments, cleaners, and air-fresheners and illustrates how scent plays a key role in delivering benefits such as malodor control, provides important in-use and post-use signals about the status of environments and objects, and makes routine home managements tasks more agreeable, motivating and helpful for facilitating social interactions. Our framework is supported by new data from a market research survey, literature in the psychological, neuroscientific, and physiological study of olfaction, as well as the fields of scent and sensory marketing. We also discuss the importance of scent for general health and wellbeing; the implications of which can be applied across many disciplines. For example, a key insight from the discussion of olfaction and health is the importance of continued exposure to a diverse olfactory environment to maintain long-term olfactory, physical, and cognitive function. In the realm of consumer products, our discussion provides a nuanced and deeper understanding of the importance of congruence, novelty, and cross-modal interactions on consumer perception and receptivity to scents, all of which lead to suggestions for future innovations in basic olfactory research as well as product development and marketing.

## Introduction

From candles to cleaners most consumers prefer scented over unscented versions of common household products, and this preference is reflected in purchasing behavior. In the United States (US) scented products represent 89% of laundry, 79% of surface cleaning, and 99% of dish washing product sales (Marmo, 2021). This is true even when unscented versions that include ideational language such as “free from” or “sensitive” are available. Indeed, most contemporary consumers consider scent an essential and necessary component of everyday household products for freshening air, laundry, and cleaning. In a research survey on consumer attitudes and preferences presented here (see Table 1 for details) consumers reported that it was “important” or “somewhat important” for scent to be added to air fresheners (97.1%), laundry products (82.1%), and household cleaners (78.2%).

**Table 1:** P&G market research survey on the purpose and preference for scent in household consumer products

Product category	Importance of scent in product for you	Percent endorsed
*Importance of scent in household products as reported by product users*		
Air freshener (*N* = 1326)	Very important	72.3
	Somewhat important	24.8
	Not at all important	2.9
Household cleaning (*N* = 1665)	Very important	41.3
	Somewhat important	36.9
	Not at all important	21.8
Laundry (*N* = 1722)	Very important	54.1
	Somewhat important	28.0
	Not at all important	17.9
Question: *How important is it for air freshening products, household cleaners, laundry care products to have a scent/fragrance?* Respondents were given a list of responses to choose from, only one response was allowed		

Product category	Main purpose of scent in product for you	Percent endorsed
*The main purpose of scent in household products*		
Air freshener (*N* = 1313)	To eliminate odors from the air	44.7
	To make my home/car smell clean	33.5
	To scent/freshen the air in my home/car	30.2
	To create a pleasant ambiance/atmosphere	29.7
	To make my home/car feel clean	20.9
Household cleaning (*N* = 1576)	To leave behind a pleasant scent after cleaning	62.2
	To remove unpleasant odors from surfaces	35.9
	To provide a pleasant experience while cleaning	27.5
	To indicate that the space has been cleaned/sanitized	26.4
	To cover up any chemical odors that the cleaning product itself may have	12.8
Laundry (*N* = 1580)	To add a pleasant smell to fabrics	43.8
	To make fabrics smell clean	40.8
	To remove unpleasant odors from fabrics	38.7
	To make fabrics smell good for a long time	29.8
	To offset any chemical odors from detergent or other (e.g., bleach)	9.3
Question: *What is the main purpose of scent/fragrance in laundry care products, air freshener products, household cleaners for you, if any?* Respondents were given a list of responses to choose from, more than one response was allowed. Top five responses per category are reported. Note that the percent endorsed for each item only captures what the consumer perceived as the primary purpose of scent in a product; secondary reasons such as liking of scent are not reflected here		

Task	Sign of a job well done	Percent endorsed
*Signal of a job well done when cleaning and doing laundry*		
Cleaning your home (*N* = 1763)	When it looks clean	75.0
	When it smells clean	54.1
	When it feels clean	33.1
	Other	1.4
Washing your clothes (*N* = 1759)	When they smell clean	76.4
	When they look clean	68.3
	When they feel soft	16.0
	Other	1.3
Question: *How do you know when the job of cleaning your home, washing your clothes, or other items, is a job well done?* Respondents were given a list of responses to choose from, more than one response was allowed		

Topic	Decision to re-wash	Percent endorsed
*Decision of re-washing clothing if they don’t smell clean*		
Washed clothes don’t smell clean (*N* = 1759)	Yes	36.4
	No	31.8
	Sometimes	31.8
Question: *After you’re done with the laundry, if the clothes or other items you’ve washed don’t smell clean, do you re-wash them?* Respondents were given a list of responses to choose from, only one response was allowed		

Topic	Feeling evoked	Percent endorsed
*Feelings evoked by having a home that smells good*		
Home that smells good (*N* = 2000)	Clean	64.3
	Relaxed	54.4
	Accomplished	44.0
	Confident	39.4
	Organized	35.8
Question: *How does having a home that smells good make you feel?* Respondents were given a list of responses to choose from, more than one response was allowed. Top five responses are reported		

Product category	Amount of scent preferred	Percent endorsed
*Preference for amount of scent in products*		
Air freshener (*N* = 1326)	A lot to a whole lot of scent	57.9
Household cleaning (*N* = 1665)	A lot to a whole lot of scent	27.7
Laundry (*N* = 1722)	A lot to a whole lot of scent	40.4
Question: *In general, how much scent do you prefer the following types of products to have?* Respondents were given a scale of responses to choose from, only one response was allowed		

Topic	Emotional connection	Percent endorsed
*Emotional connection to scents in household products*		
Scents in household products (*N* = 1759)	Yes, certain air freshener scents	19.8
	Yes, certain cleaning scents	15.7
	Yes, certain laundry scents	16.4
	No	64.11
Question: *Thinking about household products (i.e. cleaning products, laundry care products, air fresheners), are there certain scents that you are emotionally connected to?* Respondents were given a list of responses to choose from, more than one response was allowed		

Product category	Feeling evoked	Percent endorsed
*Feelings evoked by emotionally connected scents*		
Emotionally connected scents in household products (*N* = 647)	Calm/relaxed	66.2
	Joyful/happy	49.3
	Reassured/comforted	46.7
	Nostalgic/reminiscent	33.2
	Energized/stimulated	27.2
Question: *How do those [emotionally connected scents from question above] make you feel?* Respondents were given a list of responses to choose from, more than one response was allowed. Top five responses are reported		

The desire for scent is not a new or modern phenomenon. Humans have altered their environments with scent for purpose and pleasure for millennia. The word “perfume” comes from the Latin “*per fumum,*” meaning “through smoke,” which reflects its most ancient use—the burning of resins and aromatic woods to scent the air during religious rituals and ceremonies (Herz, 2011). Ancient Egyptians (3000 BCE) used aromatic compounds in embalming and deposited bouquets of rosemary in tombs to anoint the journey to the afterlife (González-Minero & Bravo-Díaz, 2018). The personal wearing of scent, or perfume, has its earliest recorded use in Egyptian murals in the fifteenth century BCE; the oldest known Chinese perfume artifacts are a pair of pomanders found in a sixth century BCE tomb (Olivia, 2016; Price, 2018).

Scented household products also have a long history dating back to the incorporation of herbs and spices into cosmetics, candles, and soap. According to Classen et al. (1994) scent dominated the ancient domestic arts. Housekeepers sprinkled scented water on clean floors and cushions, scattered bedsheets with aromatic herbs, and stored clothing in cedar chests to both fragrance them and protect from them insects. The housekeepers of antiquity burned incense in doorways to prevent foul air from penetrating the home, just as modern housekeepers use air fresheners to diminish environmental malodors. The modern custom of using scented candles in the home is also presaged by the ancient custom of using scented lamp oils to disguise the base odor of the lamp oil and to literally bring scent to light.

Scientific discoveries of the nineteenth and twentieth centuries instigated a period of technical innovation that led to the first synthetic fragrances (lab-created scent ingredients) by organic chemists (Fortineau, 2004). Fragrance innovation continued in Europe with the development of new methods for extraction using various solvents (Fortineau, 2004). Advancements in the manufacturing of synthetic fragrances enhanced safety, availability, and variety and reduced the cost of scented consumer products, thus increasing access for more individuals. Prior to the twentieth century, natural fragrance ingredients were in limited supply and often prohibitively expensive, resulting in only the wealthy being able to enjoy perfumes and scented goods. Chemical synthesis has allowed for the customization of scent molecules to be compatible with the chemistry of a variety of consumer products, including detergents and cleaners (Fortineau, 2004), thus leading to the ubiquity of scented household consumer products today.

It should also be noted that though “natural” fragrances are perceived as superior by the general public (Herz, 2003a), synthetic fragrance ingredients have a number of advantages—including for the environment and human health. For example, synthetic fragrances are highly regulated, and lead to fewer allergic reactions than “natural” aromatics (Meakins, 1999). Additionally, life cycle analysis, which evaluates the environmental impact of a product, reveals that naturally derived menthol requires energy intensive steam distillation to produce and results in 5–10 times the amount of CO_2_ per kilogram than manufacturing the synthetic equivalent (Kulke, 2015; Sell, 2006). As a function of their economic, environmental, and formulation benefits, more than 95% of fragrances used in household consumer products today are synthetic (Fortineau, 2004), and the global fragrance industry is currently valued at more than 32 billion US dollars (IMARC_Group, 2021).

Given the extensive history, pervasiveness, and preference for scent in household products, there must be substantial psychological, physiological, and social benefits to their use. For example, market data from leading manufacturers shows that during times of stress, such as after the September 11, 2001 terrorist attacks on the US, and during the coronavirus disease (COVID-19) pandemic lockdown, a large increase in the purchase of scented products and preference trends toward scents that have comforting and stress-relieving associations was seen (Z. Dubois, personal communication, February 12, 2021; Repko, 2020). This illustrates the real-life emotional needs that consumers derive from scented products. However, the factors that initiate and maintain a preference for scented household goods are not readily apparent.

To explain the general consumer preference for scented household products, we propose a three-factor framework of benefits comprising (1) functional, (2) in-use experience, and (3) emotional components. To illustrate the importance of these factors and how they support our framework we present new data from a consumer survey on fragrance in household products (laundry, air care and cleaners). We further explain our framework on the basis of contemporary literature in scent psychology and neuroscience as well as studies from sensory marketing.

### Scent in household consumer products: a three-factor benefits framework

**Fig. 1:**
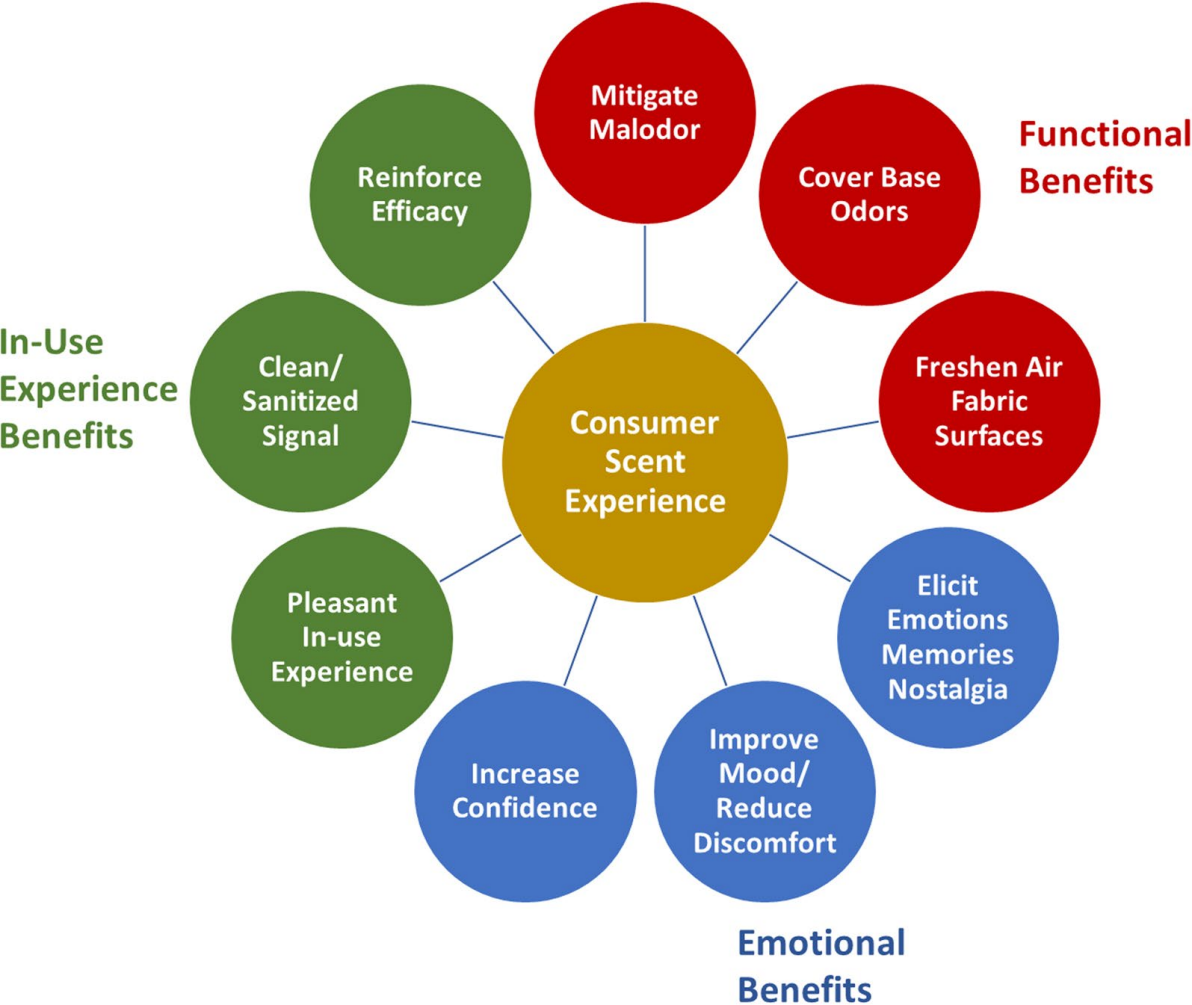
Three-factor benefits framework for scent in household products

The goal of the fragrance designer is to create a product that engages the consumer not only with cognitive and functional product attributes, but also with aesthetic, emotional and behavioral outcomes including decision making, purchase, usage, and after-use experiences (Fenko & van Rompay, 2018; Fernandes & Moreira, 2019; Oliver et al., 1997). Based on this foundation we propose that the role scent plays in consumer use, and the preferences witnessed for scented household consumer products (cleaning, laundry, and air freshening products) can be represented as a three-factor framework representing *Functional Benefits*, *In-use Experience Benefits*, and *Emotional Benefits* through which the consumer interacts with and experiences scented household consumer products (see Fig. 1). Our framework is supported by a market research survey study presented in the next section.

## Market research survey and three-factor benefits framework

### Overview of survey aims, methods and results

The aim of this survey was to gather new data with US consumers on scent preference and the perceived purpose of scent in household products. The survey, designed and analyzed by the Procter and Gamble company (P&G), was conducted online in May 21, 2021 by an independent research firm, Ask Your Target Market (AYTM), and involved 2000 US respondents who had purchased and used laundry, cleaning, and air freshening products in the previous six months. Prior to administration, the survey underwent thorough internal review, was approved by P&G legal and administrative departments, and was then pilot tested by AYTM to ensure functionality. Respondents accessed the survey via an electronic device, such as a smart phone, tablet, or computer. The survey consisted of nineteen questions, which included single-select, multi-select, open-ended responses, and/or a combination thereof. Respondents were allowed as much time as they needed to complete the survey and were financially compensated for their participation. They did not know that P&G was involved in the study in any way. Table 1 presents the key survey questions and responses, and illustrates the functional, in-use experience, and emotional benefits of scented products that underlie consumer behavior.

In the following sections we discuss the specific components of our new three-factor benefits framework and how our survey findings conceptualize and explain the scented product consumer experience.

### Functional benefits

A product’s scent must first functionally support and reinforce the task goal of the product. For example, a key task goal for scented laundry, cleaning, and air freshening products is to eliminate malodors and freshen household environments. These laundry, cleaning, and air freshening products contain scent-based technologies designed to capture or alter the molecular structure of the underlying malodor molecules, prevent perception of malodor, and/or mask the odor with fragrance ingredients, thus helping consumers feel that they are able to eliminate and control malodors (Joulain & Racine, 1989; Kato et al., 2016a, 2016b; Namba et al., 2016; Woo et al., 2011). As seen in Table 1, consumers confirm that eliminating unwanted odor is a primary reason for using scented air fresheners (44.7%), household cleaners (35.9%) and laundry products (38.7%).

In addition to direct positive benefits, the addition of pleasant fragrance reduces the negative impact on wellbeing that malodors can produce (Dalton et al., 2020; Otto et al., 1992). For example, removal of malodor has been shown to increase performance and improve subjective responses in employees, which underscores the role that odors can play in workplace productivity (Clements-Croome, 2008; Dalton et al., 2020; Wargocki et al., 2004). Several studies have also shown the ability of air freshener technologies to reduce malodors in public toilets in low-income communities, as these malodors are a reported impediment to usage (Bossut et al., 2019; Chappuis et al., 2015).

### In-use experience benefits

The second feature of a scented product’s benefit is in management of the in-use experience—providing important first impressions, improving or making the task pleasurable, and acting as a post-use signal of long-lasting efficacy, such as the scent of laundry indicating that clothes are clean (Fenko & van Rompay, 2018). The potential for product scents to reinforce the intended use of the product and improve product ratings has been well documented. Demattè et al. (2006a) showed that participants rated cotton fabrics treated with pleasantly scented fabric softener as significantly softer than fabrics treated with a softener with a less pleasant scent. Fragrance also aids the consumer in completing their tasks either through encouraging proper usage or signaling when a task is complete. For example, Holland et al. (2005) demonstrated that a scent congruent with a cleaning task (in this case citrus scent and cleaning an office environment) made the concept of the task more cognitively accessible, and increased actual cleaning behavior.

Additionally, scents in household consumer products provide signals to indicate that clothing, objects, as well as personal home and public spaces have been cleaned and sanitized or prepared to welcome guests (Crouse, 2010; Pink, 2005). This benefit can be seen in-use as a signal that the product is working and post-use as a signal that the item is still clean. Indeed, 54% of US consumers surveyed here said that a cleaning job is “well done” when the home smells “clean”. Similarly, fragrance is a critical signal for clean laundry when consumers smell clothing and fabrics to assess their cleanliness status; that is, for the absence of body odor and the presence of a fresh or pleasant scent (Pink, 2005). More than 76% of consumers surveyed here stated that smell was the signal of a job “well done” when cleaning clothing, which was significantly (95% CI) higher than the signal they obtained from visual inspection (68%). Even more striking, 36.4% of consumers stated that they will rewash clean clothing if it does not smell clean. Thus, the scent in various household products provides critical information with implications that help avoid wasting time, effort and resources (e.g., water, electricity).

### Emotional benefits

The third pillar of scented product benefits is in the emotional responses the product elicits, which in turn support belief in the product’s deliverables and increase affiliation to the product brand (Bone & Jantrania, 1992; Errajaa et al., 2020; Sugiyama et al., 2015). Many consumers report that a lingering pleasant scent is a primary purpose of having fragrance in their household products (see Table 1). In the laundry detergent domain, an earlier study where the facial expressions of loyal users of a scented detergent (consumers who had only used that brand in the past 12 months) were coded while they smelled the detergent or listened to a favorite piece of music found that more positive emotion was elicited by the laundry scent than by a favored musical selection (Procter & Gamble, 2014, internal report).^1^ Another emotional benefit of scent is its ability to elicit feelings of comfort and security especially when stress is experienced  (Warren & Warrenburg, 1993), and as mentioned previously, during periods of socio-cultural upheaval a substantial increase in scented product sales is seen. During the COVID-19 quarantine period, one US manufacturer of laundry products found that sales of washing machine ‘scent beads’ grew by 26%, and shares of scented laundry detergent rose by 4.7%, while, by contrast, shares of unscented laundry detergent fell by 2.1% (Johnson, 2021). Additionally, for many people, fresh smelling clothing is an important part of the emotional management of public self-presentation (Pink, 2005). As will be discussed in detail in the following section, the presence of pleasant scents can enhance mood and self-confidence, which has direct implications for scented product usage (Herz, 2003b, 2009; Knasko, 1992, 1995; Kontaris et al., 2020). More than half of US consumers surveyed here reported that having a home that smells “good” made them feel relaxed (54.4%), accomplished (44.0%), and confident (39.4%).

Given the power of scent to impact mood, it is not surprising that there is a well-defined subset of consumers who are identified by the fragrance industry as “scent seekers.” As shown in Table 1, a substantial number of product users claim they prefer “a lot” or “a whole lot” of scent in their air fresheners (57.9%), laundry products (40.4%), and cleaning products (27.7%). This is not a phenomenon unique to the US. Similar studies in Europe have reported that the “scent seeking” consumer segment for laundry products is also relatively large; UK (57%), France (39%), Germany (39%), and Italy (64%) (Bernaud, 2021, Proctor & Gamble, unpublished report).

As discussed in detail in the following section, the power of scent to evoke emotional memories is one of the defining features of olfaction. In the present survey of air freshener, laundry, and household cleaning products, over one-third (35.9%) claimed to have an emotional connection to the scents found in these products. Respondents further endorsed that familiar and favorite scents left them with a variety of positive emotions including “calm” (66.2%), “happy” (49.3%), and “comforted” (46.7%).

Product scents that are associated with meaningful or emotional events become both a product scent signature and a cue that elicits happy and nostalgic memories. For example, parent-infant bonding is a critical emotional milestone, and odors associated with infants has been shown to stimulate brain reward centers in women, especially new mothers (Lundstrom et al., 2013). Thus, it is not surprising that the scents in laundry products designed for baby clothes have become symbolic with babyhood. In a survey for a leading US detergent designed for baby clothes taken by 2000 new parents, 90% said that the scent itself reminded them of “baby”, and 87% said that the scent made them feel more connected and closer to their newborn (McGrath, 2018).

## A focused review of olfactory perception and psychology as foundation and support for the three-factor benefits framework

Olfaction occurs when volatile odorant molecules enter the nose as we sniff, breathe, and chew food (Stockhorst & Pietrowsky, 2004). The odorants then connect with the mucous membrane covering the olfactory epithelium and are transported to the dendrites of the olfactory sensory neurons (OSNs) where chemical stimulation is converted to an electrical signal. The OSNs extend from the epithelium through the cribriform plate, a porous bony structure separating the nasal cavity from the brain, and from there connect to the olfactory bulbs at the base of the  frontal lobes. Of high relevance for olfactory processing is the proximity of the olfactory bulbs to the amygdala-hippocampal complex. The amygdala, where emotion and emotional memory is processed, and the hippocampus, which is involved in associative learning and various forms of memory and spatial organization, comprise the primary olfactory cortex, That is, in contrast to all other sensory inputs, olfactory information is relayed first and foremost to brain areas subserving emotion, learning and memory (Cahill et al., 1995; Herz et al., 2004a, 2004b; Poellinger et al., 2001; Savic et al., 2000). The unique neural architecture of the olfactory system underlies the instantaneous responses that odors elicit which affect emotional states, arousal levels, and cognitive processing (Kontaris et al., 2020). This immediacy of emotional responding in olfaction also helps explain the role that scent plays in signaling safety and cleanliness in environments and objects—a key in-use and post-use benefit for scented household products such as laundry, cleaning, and air fresheners.

The sense of smell is greatly underappreciated by the average person (Wrzesniewski et al., 1999; Herz & Bajec, 2022), though it fundamentally underpins most experiences of daily life. Importantly, olfaction is deeply involved in psychological and physiological states that guide behavioral decision making that can impact consumer choice. The mechanisms underlying these olfactory effects are derived from the acquired emotional responses that scents elicit, which then generate psychological and physiological outcomes (Herz, 2009).

In the following sections we present a brief targeted review of olfactory research illustrating the importance of scent in daily life and its critical role in memory, mental and physical wellbeing, motivated behavior, and social behavior, and point to how the impact of scent on these processes supports our three-factor benefits framework and elucidates consumer responses to scented products. A comprehensive review of the topics discussed below is beyond the scope of the present article. For some examples of more detailed examinations the reader is referred to Herz (2016), Kontaris et al. (2020), Larsson and Willander, (2009), and Stockhorst and Pietrowsky (2004).

### Impact of scent on memory

It has been well documented that autobiographical memories evoked by odors (i.e., the Proustian memory experience) are different from memories evoked by sight and hearing (Arshamian et al., 2013; Herz, 1998, 2016; Herz & Schooler, 2002; Larsson et al., 2014; Toffolo et al., 2012). From reviewing the behavioral and neuroanatomical findings on autobiograhical memories evoked by the sense of smell, Larsson et al. (2014) determined that the key features of odor-evoked memory can be referred to by the LOVER acronym—Limbic, Old, Vivid, Emotional, and Rare. Specifically, brain imaging studies have shown that odor-evoked memories produce greater activation in the amygdala and hippocampus compared to memories evoked by visual stimuli (Arshamian et al., 2013; Herz et al., 2004a, 2004b). Odor-evoked memories are typically older then memories elicited by other sensory cues and tend to cluster in childhood (< 10 years of age) (Larsson & Willander, 2006). They are often described as vivid, and accompanied by feelings of nostalgia and a sense of being brought back in time (Chu & Downes, 2000; Herz, & Schooler, 2002; Willander & Larsson, 2006). Finally, compared to general olfactory associations, odor-evoked autobiographical memories are rare (Rubin et al., 1984; Willander & Larsson, 2007). Scent-evoked memory is a critical feature of our three-factor framework because it is through scent-evoked memory that the emotional and conceptual associations of a product are elicited which in turn reinforce and prime in-use experiences, emotional enhancements, and perceived functional performance.

### Impact of scent on wellbeing

A large scientific literature has illustrated the beneficial effects of scent on general wellbeing (Herz, 2009, 2016; Kontaris et al., 2020; Spence, 2020b). Pleasant ambient odors tend to improve mood and unpleasant odors worsen mood (Haehner et al., 2017; Knasko, 1992, 1995). People who experience odor-evoked nostalgia report higher levels of positive affect, self-esteem, and optimism (Reid et al., 2015). Pleasant scents reduce anxiety in people undergoing stressful procedures and tasks, and can even help curb cigarette cravings and other unwanted urges (Firmin et al., 2016; Hedblom et al., 2019; Lehrner et al., 2005; Sayette et al., 2019). Moreover, odors that evoke pleasant memories are associated with a reduction in physiological stress biomarkers such as respiration rate and heart rate (Campenni et al., 2004; Masaoka et al., 2012), and may boost immune system functioning (Matsunaga et al., 2011, 2013).

Exposure to scent has also been shown to be useful in alleviating discomfort and managing the perception of pain. While pain typically results from a physical stimulus, the experience of pain is strongly affected by psychological factors such as mood, emotion, and attention (Arntz & de Jong, 1993). Research has shown that the negative affect associated with pain can also be alleviated by pleasant odors (e.g., Prescott & Wilkie, 2007; Riello et al., 2019; Villemure et al., 2003; Villemeure & Bushnell, 2009). For example, subjects exposed to a preferred pleasant scent while undergoing painful heat reported improved mood, decreased anxiety, and decreased rating of pain unpleasantness (Villemure et al., 2003). Neuroimaging has confirmed that pain and odor stimulate similar regions of the brain (Villemure & Bushnell, 2009). The implications of these findings are that, among the ways that scents contribute to increased consumer satisfaction with products used in routine or mundane chores such as cleaning or laundering clothes, is through their ability to reduce discomfort and enhance wellbeing. This illustrates both the in-use and emotional benefits of scented products and suggests why consumers prefer them.

### Impact of scent on motivated behavior

The unique connection between olfaction and emotion is further witnessed in motivated behavior (Epple & Herz, 1999; Herz et al., 2004a, 2004b). Positive mood has been shown to increase productivity, decision making, and creativity (Isen et al., 1991; Politis & Houtz, 2015). Physical stamina can also be enhanced. In one study, athletic young adults ran faster and did more push-ups in the presence of peppermint aroma, and a similar study found that peppermint aroma enhanced self-evaluations of vigor and perceived performance when running on a treadmill. (Raudenbush, 2002; Raudenbush et al., 2001). Relatedly, scents help reduce feelings of fatigue and improve performance when undergoing tiring or difficult cognitive tasks (Ho & Spence, 2005; Raudenbush et al., 2009; Saito et al., 2018). The US military has even reported that introducing a scent during training exercises improved later performance when the scent was present again during subsequent tests (Vergun, 2016). Together these findings suggest that the preference for scented products used in household chores like cleaning or laundry may be partly because the scents make users feel more invigorated and the tasks less fatiguing. The ways in which scent increases motivation and propels behavior illustrate  how scent scaffolds the in-use and emotional benefits that consumers experience  from scented household products.

### Impacts of scent on social behavior

Sensory environmental cues enhance interpersonal dynamics, as well as pro-social, and cooperative behavior (Forgas, 1998). Experimental studies have found that pleasant ambient scents improved agreeableness and several measures of work cooperativity (Baron & Bronfen, 1994; Marchlewska et al., 2016). A familiar scent highly associated with cleanliness has even been shown to promote prosocial behaviors, such as reciprocating trust and charity (Liljenquist et al., 2010). These findings imply that if pleasantly scented cleaning products are used in the workplace; employees might be friendlier and more cooperative, which in turn may improve work productivity and outcomes.

When choosing personal scents, individuals are creating their sensory self-image—who they are or want to be perceived as (Spence, 2021b). In a survey of female fragrance shoppers, 82% believed that wearing perfume made them more appealing to others and 52% specifically noted that it made them more attractive (Herz, 2003a, 2003b). Many studies have confirmed that scent can influence evaluations of likeability and attractiveness from  others (Baron, 1981; Demattè et al., 2007a, 2007b; Li et al., 2007).

Importantly, it has been found that the use of scent can make people feel more confident. For example, Herz (2003b) reported that 65% of the women surveyed reported feeling more confident when they wore perfume. Improved self-confidence can also alter non-verbal behavior that influences perceived attractiveness. In a study where men were videotaped when they either wore a scented deodorant body spray or not, women rated the men as more attractive when they were wearing scent purely on the basis of the men’s body language in the video clips (Roberts et al., 2009). In another study, video observers rated women wearing perfume as more confident, and video analysis confirmed that there  were fewer anxious behaviors such as face touching and fidgeting when the women wore fragrance (Higuchi et al., 2005). The scent of clothing further   contributes to attractiveness perception. Kerr et al. (2005) found that a hypothetical person  whose clothing was  associated with a canoically clean scent was rated as more intelligent, attractive, successful and sociable than someone  whose clothing was associated with a scent that was not synonymous with  clean. Thus, "clean" scented laundry products both  increase positive perception by others and enhances the self-confidence of users which then can promote social behavior that leads to increased perceived attractiveness (e.g., Roberts et al., 2009). The role of scent in social behavior showcases how all three components of our framework; functional, in-use experience, and emotional, operate together.

**Fig. 2:**
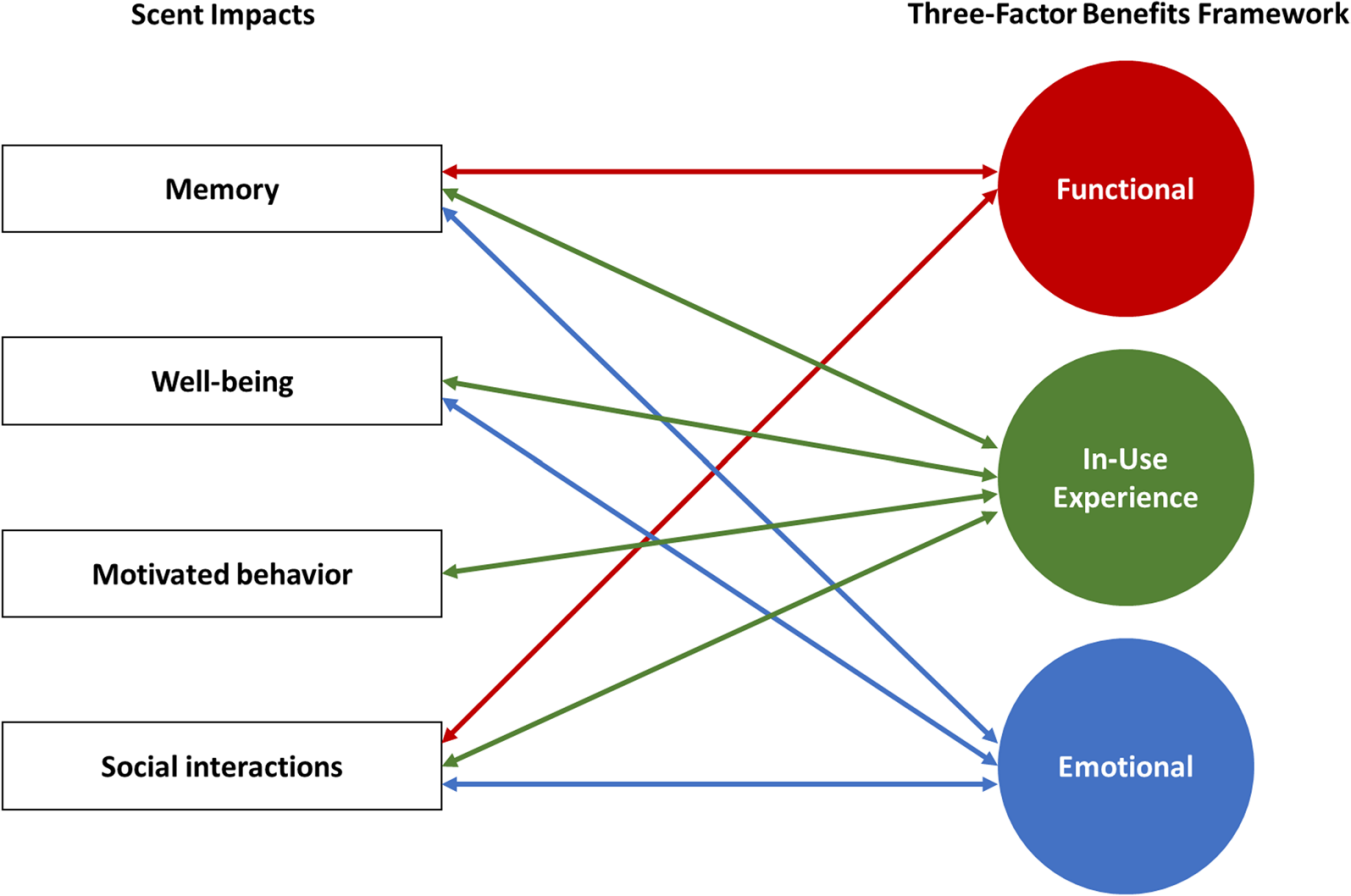
Bidirectional interactions between scent impacts and consumer benefits in the three-factor framework

Figure 2 provides a schematic illustration of how the impacts of scent on memory, wellbeing, motivated behavior, and social behavior that have been discussed here bidirectionally interact with the consumer-product experience in our three-factor benefits framework.

## Importance of fragrance for quality of life and olfactory health

Following from our discussion of the relationship between scent impacts and consumer product experiences, is how scent is involved in health and quality of life (QOL) more generally, several facets of which are relevant to our three-factor benefits framework. Central variables affecting olfactory perception include age, sex, health (e.g., vascular factors, diabetes), genetic profile (e.g., ApoE4), and clinical conditions (e.g., depression) (Larsson et al., 2000; Seubert et al., 2017). Thus physiological characteristics of the consumer can impact their perception and responses to scents. More extreme examples of altered olfactory processing are witnessed in various forms of olfactory dysfunction (OD). OD encompasses the conditions of absent function (anosmia), reduced sensitivity (hyposmia), distorted perception (parosmia), and phantom perception (phantosmia) (Croy et al., 2014). OD can arise through various causes including viral infection, head trauma, nasal obstruction, and exposure to toxins/drugs (Croy et al., 2014).

OD has a substantial negative impact on wellbeing and QOL (Burges Watson et al., 2021), the most common complaint of which is decreased enjoyment of food, which can lead to changes in weight and in some cases impaired nutrition (Boesveldt et al., 2017; Croy et al., 2014). Anosmia also incurs an added risk for exposure to hazards such as fire, natural gas, and food poisoning (Pence et al., 2014). A substantial number of people with long-term smell loss exhibit depressive symptoms, diminished self-esteem, and a loss of intensity of emotional experiences (Croy et al., 2014; Herz, 2000; Kollndorfer et al., 2017; Smeets et al., 2009). A particular area of maladaptive emotional responding for people suffering with OD is seen in social interactions, where concern over personal body odor can produce fear of interpersonal relationships thus leading to social isolation and withdrawal that exacerbates  other negative emotional states (Boesveldt et al., 2017; Croy et al., 2014).

Issues of personal hygiene have particular relevance for our three-factor benefits framework. For example, consumers with OD can be reassured that household products with pleasant fragrances will make their clothes smell clean, and the air fresheners and cleaners they use in their home will mitigate potential malodors. Thus, scented household products can increase confidence and lessen anxiety about socializing and entertaining at home, which may help overcome social withdrawal.

A metanalysis of OD studies conducted between 1992 and 2019 found that the pooled prevalence rate of any kind of impaired olfaction was 22.2% (Desiato et al., 2021). Notably this meta-analysis was conducted on data collected prior to COVID-19. A major symptom and long-term consequence of COVID-19 is smell loss. A study published in the *Journal of the American Medical Association* in November 2021 reported that as many as 1.6 million people in the US alone were suffering from long-term (six months or greater) OD resulting from COVID-19 (Khan et al., 2021). Thus, a non-trivial and currently growing segment of the consumer population has impaired olfaction. How this will impact purchasing behavior of scented products is not yet known, but if marketers can convey the psycho-social benefits of scented household products to customers—especially as pertains to quality of life and olfactory health–it may help to increase usage.

Encouragingly, a distinctive feature of the olfactory system is its plasticity and capacity for neuronal regeneration (Beecher et al., 2018; Schwob, 2002). Research has further shown that olfactory plasticity is promoted by odor stimulation, and a dysfunctional sense of smell can be restored in many individuals by deliberate sniffing and smell training (Sorokowska et al., 2017). In fact it has been found that smell training can produce functional and structural reorganization of olfactory brain areas (Al Aïn et al., 2019), and may facilitate transfer of learning to other sensory domains (Olofsson et al., 2020). This is of critical significance for the availability of scented consumer products, as it underscores the value of having a rich and diversely scented environment and suggests that scented consumer products may indirectly support mental and physical health.

## Marketing principles and implications for scented consumer products

### Scent marketing

Our three-factor benefits framework is a theoretical tool that can be used to inform and inspire marketing professionals and product developers. By extension the framework has particular application to “scent marketing”, in which scents are used to enhance product perception, purchasing behavior, and consumer responses. Scent marketing manipulations are either ambient (in the environment where the product is judged) or intrinsic to the product (a component added to the product). A number of studies have found that ambient scent can lead to improvements towards both environmental and product evaluations (Bosmans, 2006; Fiore et al., 2000; Mitchell et al., 1995; Spangenberg et al., 1996). Ambient scent has been shown to improve the shopping experience in retail environments leading to more positive ratings for the store and increased intention to return to it (Spangenberg et al., 1996). Orth and Bourrain (2005) further found that scent-induced nostalgic memories were positively related to consumer sensation seeking and curiosity-motivated behaviors.

Utilizations of ambient scent also yield positive results beyond retail settings. In the hotel industry, having a lobby scented with a signature fragrance improves guest ratings of cleanliness and comfort (Crouse, 2010). A recent study conducted in Portugal found that introducing an ambient scent in a passenger bus resulted in more positive memories of the travel experience and increased intentions to reuse or recommend the bus service (Silva et al., 2021). In Singapore, adding air freshening scents to city buses was implemented to reduce tension among riders and increase intent to use public transportation (Poon, 2017; Spence, 2021a). Thus, scent marketing can facilitate the in-use experience and provide emotional benefits in numerous public spheres (Minsky et al., 2018).

With respect to scent added to products, one early study reported that the addition of a pleasant floral scent to silk stockings enhancedconsumer preference for the scented over unscented stockings (Laird, 1932). More recently, it was shown that body lotion scented with a fragrance that the user found pleasant and personally evocative led to the lotion being rated more positively on all the functional (e.g., “provides long-lasting moisturization”) and emotional (e.g., “makes me feel good when I use it) attributes of the lotion that were assessed (Sugiyama et al., 2015). These examples illustrate how scent marketing can increase both functional and emotional benefits of various products.

As an example of how memory interacts with our three-factor framework, Krishna et al. (2009) presented pencils to consumers that were either scented with a common smell (pine), an uncommon scent (tea tree) or unscented, along with lists of their respective attributes, and found that recollection was highest for the information related to either of the scented pencils compared to recall for the unscented pencil. Relatedly, Morrin and Ratneshwar (2003) found that subjects exposed to ambient scent while viewing product photos had higher 24-h brand recall and recognition accuracy.

Importantly, to positively influence consumers’ product evaluations, their mental and emotional associations need to be congruent (consistent with the pre-existing cognitive schema) with expected product attributes (Bosmans, 2006; Errajaa et al., 2020). Multiple studies have found that congruency makes it easier for consumers to process information, which results in faster recognition and more positive product evaluation (Brod et al., 2015; Meyers-Levy & Tybout, 1989). In retail settings, a scent that is perceived as congruent with the product theme and other store cues leads to increased purchase behavior and sales (Mattila & Wirtz, 2001; Spangenberg et al., 1996). Bone and Jantrania (1992) also found that consumer ratings of sunscreen and household cleaner were improved when the product scents were congruent with consumer expectations of the product category—coconut sunscreen and lemon cleaner were preferred over incongruent pairings of lemon sunscreen and coconut cleaner. Notably, when a pleasant scent is perceived as incongruent with a product in context, it can lead to a marked decrease in perceived value, purchase intention, and future sales, and can be  substantially more negative than having no fragrance at all (Fiore et al., 2000; Herz, 2010).

### Sensory marketing and cross-modal effects on product perception

Beyond the utilization of scent to invigorate product perception and purchasing behavior, is the general field of sensory marketing where multiple sensory features of a product are capitalized upon for their interactions in augmenting product perception. For example, it was recently shown that scent enhanced the texture/tactile properties of a cosmetic lotion; with added scent, lotion liking, texture liking, and ratings of wellbeing from usage all increased (Courrèges et al., 2021). Thus, in order to fully understand the impact of scent on product perception, it is important to adopt a multimodal perspective. Specifically, as we process our environment through multiple perceptual modalities simultaneously, it is crucial to further our understanding of how scent may interact with other sensory modalities to influence consumers experiences and behavior. As noted by Schifferstein (2006), the relative importance of each modality depends on the type of product and on the type of evaluation. For instance, scent perception is more informative than vision for determining whether a product is safe or clean.

It is well known that vision is  the dominant sense in human information processing, and most studies on cross-modals interaction have focused on the interplay between olfaction and vision. Several findings related to product perception indicate that color affects and biases how we perceive and identify scents. For example, Morrot et al., (2001) reported that when presented with a white wine that was colored red, wine experts used descriptors that were typical for red wines. Likewise, although specifically instructed to ignore visual information (color) that was jointly presented with a scent, Demattè and colleagues (2006b) found that identification responses were biased to the color. Similar bias effects were reported for perceived scent intensity that varied as a function of the degree of visual lightness (Kemp & Gilbert, 1997).

Less is known about cross-modal interactions of scent with other sensory modalities. With a focus on scent and auditory input, Crisinel and Spence (2012) examined the relation between odor quality and auditory pitch and found that fruity scents were related to higher pitch. In a similar vein, Spangenberg et al. (2005) highlighted the important role of semantic congruence between scent and information obtained through hearing. Here, the combination of a congruent scent and music (Christmas scent—Christmas music) as compared with the same scent and non-Christmas music in a store yielded a better shopping experience and higher retail profitability.

The available evidence also supports the existence of cross-modal interactions between olfaction and touch. For example, Demattè et al. (2006a) reported that fabric swatches were perceived as softer when presented with a lemon scent than when presented with an animal-like odor. In a follow-up study, it was demonstrated that these conceptually congruent associations also emerged when assessed through implicit tests (Demattè, Sanabria, & Spence, 2007). The modification of touch perception in the context of olfactory stimulation was further demonstrated by Croy et al. (2016), who found that the sensation of pleasant touch was perceived as less pleasant when experienced in the context of a subjectively disgusting odor. In a similar vein, Churchill et al. (2009) examined whether different pleasant scents added to shampoo would affect the perception of hair after washing, and found that the different odors affected the perception of hair texture characteristics,  supporting the notion of a cross-modal interaction between scent and touch. Additionally, as mentioned earlier, scent can enhance the perception of texture in cosmetic lotions (Courrèges et al., 2021). Thus, tactile-olfactory interactions present a particularly fruitful area for future research in consumer behavior (Courrèges et al., 2021; Demattè et al., 2007a, 2007b; Krishna et al., 2010).

### Potential for negative responses to fragrance in consumer products

In spite of a majority preference for scented household products, there is a small subset of consumers who dislike fragrance in products and consider it to be a pollutant or invasion of their personal space and choice. The industry term for these individuals is “scent avoiders”, to contrast with “scent seekers” as we have discussed so far. The percentage of consumers who prefer unscented versions of products and are “scent avoiders” varies by category. For example, in hand dish washing products it is 7%; in the beauty care category approximately 5% of consumers prefer to use unscented body wash, skin creams and lotion; whereas in laundry care products only 3.7% prefer unscented formulations (P&G unpublished internal data from a 2017 study of 3505 representative US consumers).^2^ With respect to household products, focus group interviews with one of the authors of the present paper revealed that the central reasons for fragrance avoidance are health concerns, such as fear of triggering an asthmatic episode or general fear of inhalation of “chemicals”. Interestingly, this fear of chemical exposure may bear some relation to the condition of multiple chemical sensitivities (MCS).

MCS is a controversial disorder in which individuals report various physical symptoms elicited by a wide range of environmental chemicals at concentrations far below standardized levels of harm, and for which objective evidence of physical ailments is often lacking, though distress is clearly felt by the person (for an excellent comprehensive review of MCS see Zucco & Doty, 2022). Germane to the present discussion, a key trigger category for MCS is household cleaners. In this light it would be valuable to investigate the commonalities between fragrance avoiders and MCS. For example, do fragrance avoiders progress to MCS? Are there similar underlying mechanisms in fragrance avoiders and MCS (e.g., fear)? Knowledge of these mediating mechanisms could help mitigate avoidance of fragrance in products generally and potentially prevent people from developing MCS.

## Suggestions for future research and innovation

In the past several decades, empirical evidence from genetics, biochemistry and neuroimaging as well as clinical developments have improved and advanced the study of olfaction (Buck & Axel, 1991; Cahill et al., 1995; Herz et al., 2004a, 2004b; Poellinger et al., 2001; Savic et al., 2000). Nonetheless, olfactory research is underfunded and under resourced. In 2020, the National Institutes of Health budget for smell and taste research was nearly one-quarter of that apportioned for either auditory or balance studies, and there have been more than 10 times as many scientific studies published on visual memory than olfactory memory, despite the unique role of olfaction in memory (Hutmacher, 2019; Munger, 2021).

The COVID-19 pandemic has raised public awareness of olfaction and the importance of scent for health, wellbeing, and QOL. Smell training has become popularized and recent successes in smell training (Al Aïn et al., 2019; Olofsson et al., 2020; Sorokowska et al., 2017) for improving olfactory and cognitive function indicate the wide-ranging implications for  the role of scent in  health and wellbeing. Studies are now needed to elucidate the improvements that scents can have on mental and phsyical health and to broaden public knowledge of these benefits. For example, can a scent-enriched environment improve and extend cognitive and olfactory function during aging?

A central mission of the present paper is to stimulate research into how scent can be used for consumer benefits in the scented product domain. Some paths for future investigation include: determining how specific scents can alter texture perception, the interaction of scent with other product sensory attributes and verbal labeling, the impact of scent-evoked memories, and how the particular product in question influences consumer responses. For example, in numerous empirical and applied demonstrations, Spence and colleagues have reported how vision, hearing and touch can interact with and alter the perception of olfactory experiences in products and otherwise (Crisinel & Spence, 2011; Hanson-Vaux et al., 2012; Spence, 2020a, 2021b). Further investigations into how scent interacts with various product features, and how these factors may differ depending on the specific touchpoint of the product in question now needs to be investigated.

**Fig. 3:**
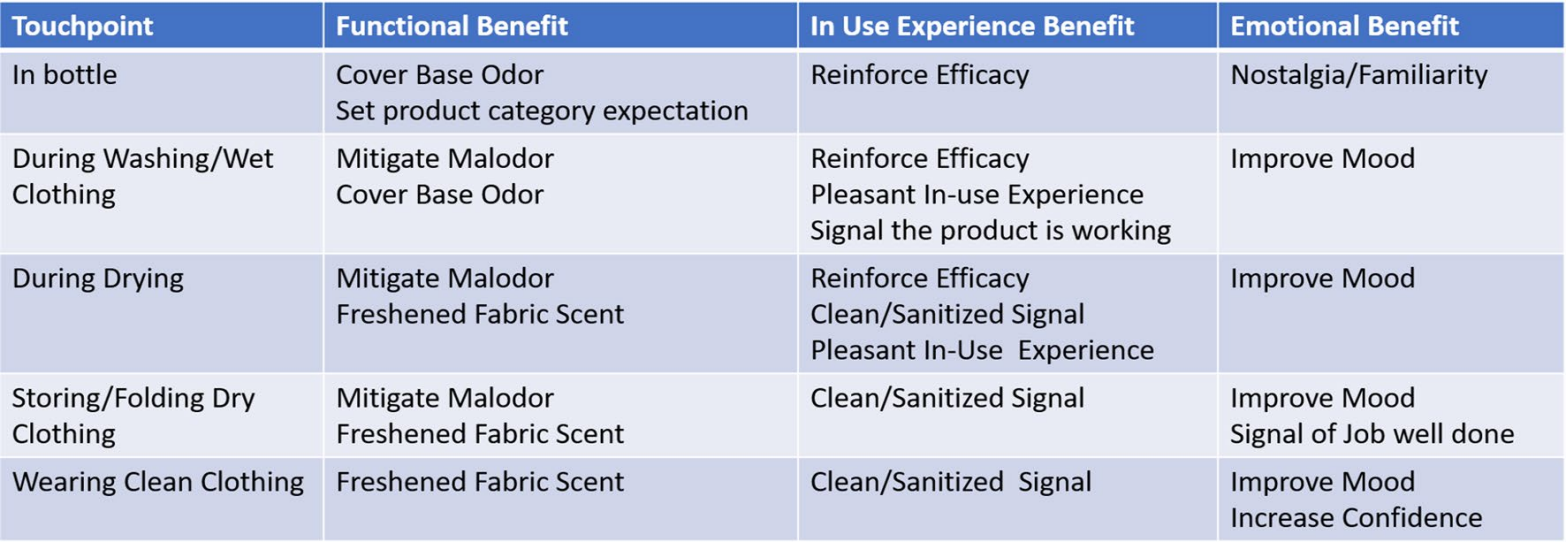
Scent benefits framework as experienced across a laundry touchpoint model

Touchpoints are the distinct points of interaction between the consumer and the product during pre-use (purchase/consideration), usage, and post-use. For example, skin for lotion; clothing for laundry; and ambient space for air-fresheners. Figure 3 shows, with the example of laundry detergent fragrance, how the lifecycle of scent touchpoints provides opportunities for benefits through: (1) the scent of the product in the bottle when opening the cap in-store to make a purchase selection and again while adding the detergent to the washing machine; (2) the scent of the wet clothing as it is moved from the washing machine into the dryer; (3) the scent emanating during the drying cycle; (4) the scent of the dry clothing while folding and storing; and (5) the lingering scent on cleaned clothing during the next wear. Notably, the touchpoints of a fragrance designed for a detergent will differ from a fragrance in an air freshener and will possess design features specifically targeted to deliver benefits while the consumer is using a particular product throughout its lifecycle.

In addition to scent quality, the intensity of a scent needs to vary at specific touchpoints of use so that it elicits and reinforces the intended product concept and enhances functional benefits. Scent pleasantness is typically related to intensity following an inverted-U function, with pleasantness increasing up to a point and then decreasing as intensity further increases (Martindale & Hines, 1975). However, at specific touchpoints in product usage the intensity and character may vary. For example, with laundry products, scent is strongest upon first exposure (bottle opening) where it elicits a powerful cleaning efficacy signal, and is less intense and more wearable in character on dry clothing so that the scent does not clash with other personal fragrance use.

An additional avenue for research is to investigate whether and how demographic factors, such as personality characteristics (e.g., being a “scent seeker”), as well as age, gender, geography, and socioeconomic status, influence what is expected and preferred in product scents. A related question is how the sensory properties of touch, sight, and sound affect consumers’ perception of a product’s scent. For example, Gatti et al. (2014) found that the color and weight of packaging altered the perceived intensity of fragrance in dish soap. In this vein it would be useful to investigate how the conceptual meaning of a product category mediates what cross-modal interactions most influence the perception of scent. That is, might verbal labels have a more profound influence on the perceived scent of cleaning products while colors are more impactful for personal care products? Responses may also vary and interact with aspects of consumer demographics. Cross-modal interactions present a rich area for further research in the scented product domain.

One of the challenges for scented product developers is to find a balance between the tendency towards the familiar and the need for novelty. Scents that harken back to personally relevant or familiar memories typically lead to positive evaluations and acceptance (Sugiyama et al., 2015), and the psychological benefits of nostalgia are well noted (Reid et al., 2015). Sensory stimuli that are consistent with a pre-existing schema, have been found to reinforce product efficacy, and these congruent scents also tend to be familiar (Demattè et al., 2006b; Errajaa et al., 2020; Meyers-Levy & Tybout, 1989). However, reliance on the familiar may be a hindrance to innovation and creativity. While many studies have shown that conceptual congruency between a scent and a product reinforce consumer responses and perceived product value (Doucé et al., 2013; Errajaa et al., 2020; Fiore et al., 2000; Meng et al., 2021; Spangenberg et al., 2006), others have found that under certain circumstances moderately incongruent scents were rated more favorably  than congruent scents due to their novelty (Bosmans, 2006). Further research into understanding scent schemas and congruency will aid product developers in creating new scents and to better understand the evolution of scent trends. This is of particular relevance for determining how and when the fragrance of a consumer product becomes iconic and when it is time to refresh the fragrance of a known brand.

The three factors of the scented product experience: *function, usage,* and *emotion*, interact to influence the consumer’s perception and responses to scented products. A product’s scent alters its perceived efficacy and emotional benefits, which are then realized throughout the usage experience of the product. Understanding how consumers use scented products, how they interact with the scent at the different touchpoints, and the “benefit” opportunities at each of these stages are topics of considerable interest to the fragrance industry.

## Conclusions

The goal of the present paper was to present a conceptual framework through which consumer preferences and the potential benefits of scented products could be understood and to explain them in terms of how they relate to basic principles in olfactory psychological science. We propose that the drivers of the preference for scented products can be conceptualized by a three-factor framework of benefits. Specifically, the reason fragrance in air fresheners, cleaning, and laundry products is considered essential to most consumers is because of their *functional* (e.g., malodor control, freshening), *in-use experience* (e.g., completion signals, improved task pleasantness), and *emotional* (e.g., relaxation, self-confidence) benefits, which together create a holistic scent-product experience.

Additionally, scented cleaning and air freshening products mitigate malodors helping to prevent physical, psychological, and even economic harms (Dalton et al., 2020). In a review of the impacts of indoor malodor, Dalton et al., (2020) reported that malodors can decrease property valuation. Just as, by contrast, real estate agents often use scent to increase the desirability of a home. Typically this is done by burning candles with pleasant and nostalgic aromas, such as apple pie. A potentially under-realized opportunity in the air freshener category may be to create more scents with nostalgic fragrance profiles.

Scented cleaning products assist the consumer in achieving their objective of cleaning and freshening their clothes and environment by reinforcing efficacy and providing “cleanliness” signals that help the consumer  know what areas have been cleaned versus those that have not (Crouse, 2010; Holland et al., 2005; Pink, 2005). Scent can elevate emotions among consumers when they are performing mundane tasks, thus making theses chores more pleasant. Scents in everyday products can also facilitate social situations by boosting self-confidence, improving mood, and creating a soothing or welcoming environment (Crouse, 2010; Field et al., 2008; Herz, 2003a, 2003b; Higuchi et al., 2005). The use of scent in commercial products and in public spaces further provide signals that have become relied upon to cue cleanliness which can reduce anxiety and increase willingness to use shared transportation and sanitation facilities (Bossut et al., 2019; Crouse, 2010; Poon, 2017; Silva et al., 2021; Spence, 2020b).

Notably, it has been shown that OD has negative consequences for many aspects of psychological, physiological, and neurological health. Thus, an environment rich in diverse olfactory input may be integral for the maintenance of a healthy sense of smell, overall wellbeing, and cognitive and physiological functioning (Birte-Antina et al., 2018; Flohr et al., 2014; Kikuta et al., 2015; Olofsson et al., 2020; Pekala et al., 2016; Reichert & Schöpf, 2018).

Scent plays an essential role in human life and culture. The uniquely direct neuroanatomical connection between the olfactory system and the emotional and memory centers of the brain enable scents to immediately alter mood and wellbeing unlike any other sensory experience. The profound perceptual, cognitive, and emotional impacts that scents elicit suggest new directions for research to determine the best ways to leverage scents for improved product perception, consumer satisfaction, and overall wellbeing. Proposed areas for future research include cross-modal interactions, congruency, and the tension between novelty and nostalgia.

## Data Availability

The datasets used and/or analyzed during the current study are available from the corresponding author upon reasonable request.

## Abbreviations

COVID-19: The disease produced by the SARS-CoV-2 virus
MCS: Multiple chemical sensitivities
OSN: Olfactory sensory neurons
OD: Olfactory dysfunction
QOL: Quality of life
US: United States of America

## Footnotes

1 For study details contact Morgan Brashear: eberhard.me@pg.com.

2 For study details contact Morgan Brashear: eberhard.me@pg.com.

## Declarations

### Open practices statement

The datasets used and/or analyzed during the current study are available from the corresponding author on reasonable request. No experiments in this study were preregistered.
